# Application of Exosomes-Derived Mesenchymal Stem Cells in Treatment of Fungal Diseases: From Basic to Clinical Sciences

**DOI:** 10.3389/ffunb.2021.736093

**Published:** 2021-09-30

**Authors:** Seyedeh Ommolbanin Ghasemian

**Affiliations:** Department of Veterinary, Behbahan Branch, Islamic Azad University Behbahan, Behbahan, Iran

**Keywords:** fungi, exosome, mesenchymal stem cell, interleukin-17, toll-like receptor 4

## Abstract

Fungal diseases such as candidiasis are some of the deadliest diseases among immunocompromised patients. These fungi naturally exist on human skin and throughout the digestive system. When the microbiota balance becomes upset, these fungi become pathogenic and potentially lethal. At the pathogenesis of fungal diseases, host immune system response is diverse. At the early stages of fungal pathogenesis such as *Candida albicans*, it was shown that these fungi use the immune cells of the host body and cause malfunction the early induction of proinflammatory cytokines of the host body leading to a reduction in their numbers. However, at some stages of fungal diseases, the immune response is severe. Despite many treatments already being available, it seems that one of the best treatments could be an immune-stimulatory agent. Some of the subsets of MSCs and exosome-derived cells, as a cell-to-cell communicator agent, have many roles in the human body, including anti-inflammatory and immune-modulatory effects. However, the TLR4-primed and IL-17+ subsets of MSCs have been shown to have immune-stimulatory effects. These subsets of the MSCs produce pro-inflammatory cytokines and reduce immunosuppressive cytokines and chemokines. Thus, they could trigger inflammation and stop fungal pathogenesis. As some biological activities and molecules inherit elements of their exosomes from their maternal cells, the exosome-derived TLR4-primed and IL-17+ subsets of MSCs could be a good candidate for fighting against fungal diseases. The applications of exosomes in human diseases are well-known and expanding. It is time to investigate the exosomes application in fungal diseases. In this review, the probable role of exosomes in treating fungal diseases is explored.

## Introduction

### Host-Fungi Interactions: Normal Flora or Pathogen?

There are fungi in the human body that are known as normal flora (Prasad, 2007). This population of fungi is called fungal microbiota or mycobiota (Limon et al., 2017). Knowing these microbiotas, including mycobiota, is an important factor in host diseases and health (Limon et al., 2017). For many reasons, when the balance of these mycobiota is upset they can become a pathogen. Fungal diseases effect a quarter of the human population worldwide (Brown et al., 2012). However, while most of the fungal diseases are related to superficial skin conditions and can be treated locally, the systemic fungal infection could be so lethal (Brown et al., 2012; Vallabhaneni et al., 2016). These systemic fungal diseases usually occur because of diverse immune responses; especially in patients with immune system suppression (Pappas et al., 2018). There are lots of treatment option for systemic fungal diseases, but using them has limitations and usually brings poor outcomes (Scriven et al., 2017). It seems that one of the best choices to treat fungal diseases is reversing immune deficiency, which occurs in patients with immunosuppression (Scriven et al., 2017).

### Pathogenesis of Fungi and Host Immunity

A previous study on *C. albicans* revealed that the host immune response to *C. albicans* is downregulated at early stages by pathogenic fungi (Halder et al., 2020). It was shown that the *C. albicans* attached to the C3 receptor of the monocytes by its β-glucan. Using this attachment to the monocytes, the fungi stimulate the monocytes to release extracellular vesicles contained transforming growth factor (TGF)-β. Using TGF-β-transporting vesicles, the fungi reduce immune response and cause anti-inflammatory effects at the early stages of fungi pathogenesis (Halder et al., 2020). Moreover, using TGF-β production, the fungi could reduce early production and induction of pro-inflammatory cytokines (Netea et al., 2002; Halder et al., 2020). This is how the fungi downregulate the host immune system in order to favor its existence and survival.

### Mesenchymal Stem/Stromal Cells (MSCs), Immunosuppressive or Immune-Stimulator?

The MSCs are the progenitor/stem cells that have the capacity to differentiate into multilineage cells (Billing et al., 2016; de Castro et al., 2019). Due to their potential for differentiation, their immunomodulatory effect, and their regeneration capacity (Zhang et al., 2020a; Oh et al., 2021), they are widely used in treating injuries and some inflammatory disorders (Zhang et al., 2020a; Liao et al., 2021). Clinical studies have shown that because of the immunomodulatory function of some subsets of MSCs, MSC therapy could suppress the immune system and treat inflammatory and autoimmune diseases (Nauta and Fibbe, 2007; Yang et al., 2013). In detail, the MSCs, directly or indirectly, affect T cells and regulate them. The MSCs produce some chemokines and cytokines such as interleukin 10 (IL-10), prostaglandin E_2_ (PGE_2_), nitric oxide (NO), TGF-β, indoleamine 2,3-dioxygenase (IDO), tumor necrosis factor-inducible gene 6 (TSG-6), and chemokine ligand 2 (Batten et al., 2006; Nauta and Fibbe, 2007; Yang et al., 2013). These molecules affect CD4^+^CD25^+^ regulatory T (T reg) with positive transcription factor Foxp3 and T helper 17 (Th17) cells' population and regulate them (Batten et al., 2006; Park et al., 2011; Yang et al., 2013; Bi et al., 2020). That's how MSCs downregulate the immune system in inflammatory and autoimmune diseases.

**Figure 1:**
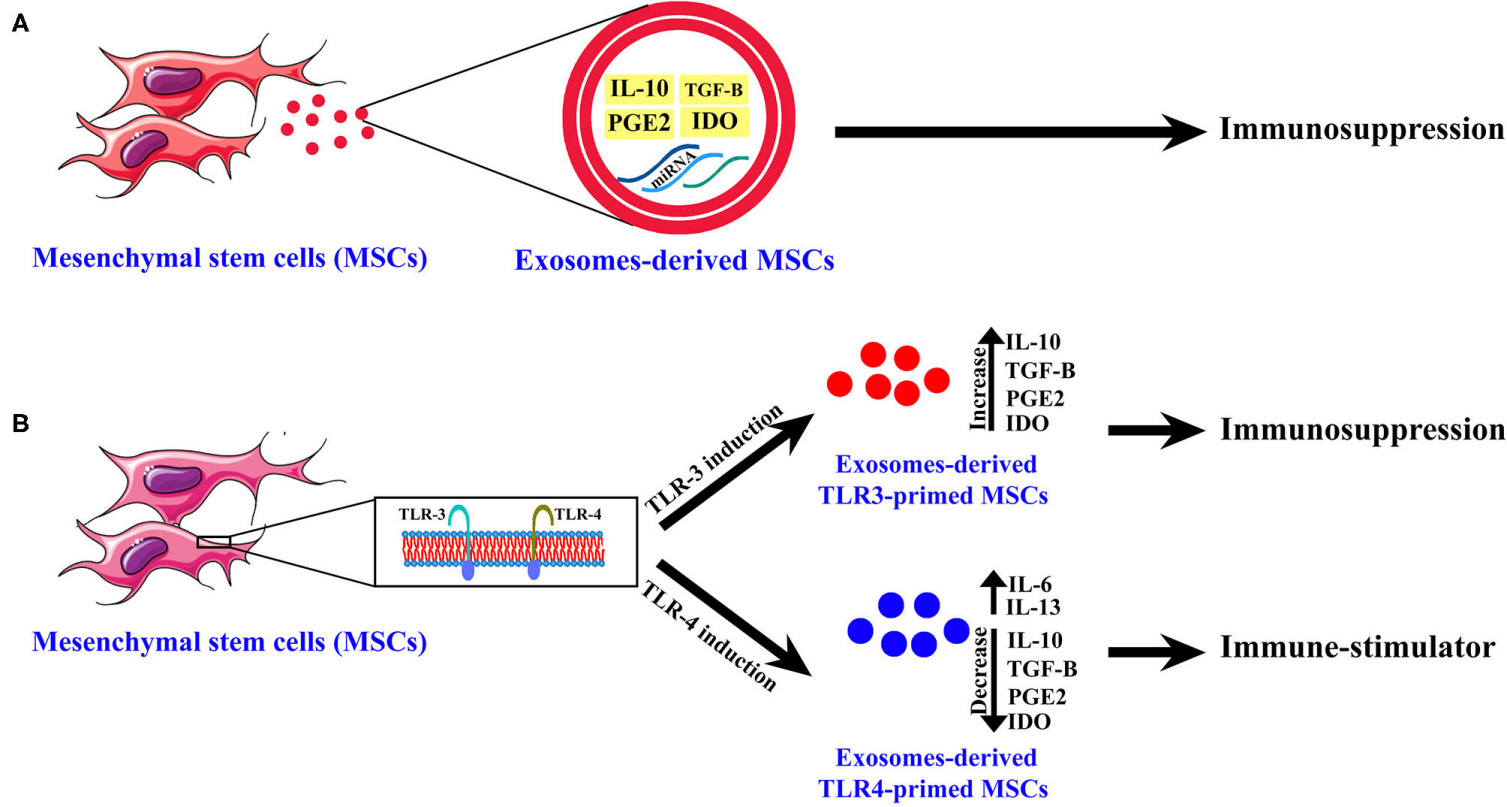
The exosome-derived mesenchymal stem cells (MSCs) cytokines and chemokines content. **(A)** Normal MSCs. **(B)** TLR3-primed and TLR4-primed subtypes of MSCs. This figure shows chemokines and cytokines of exosomes-derived MSCs of different subtypes of MSCs and their biological activity.

**Figure 2:**
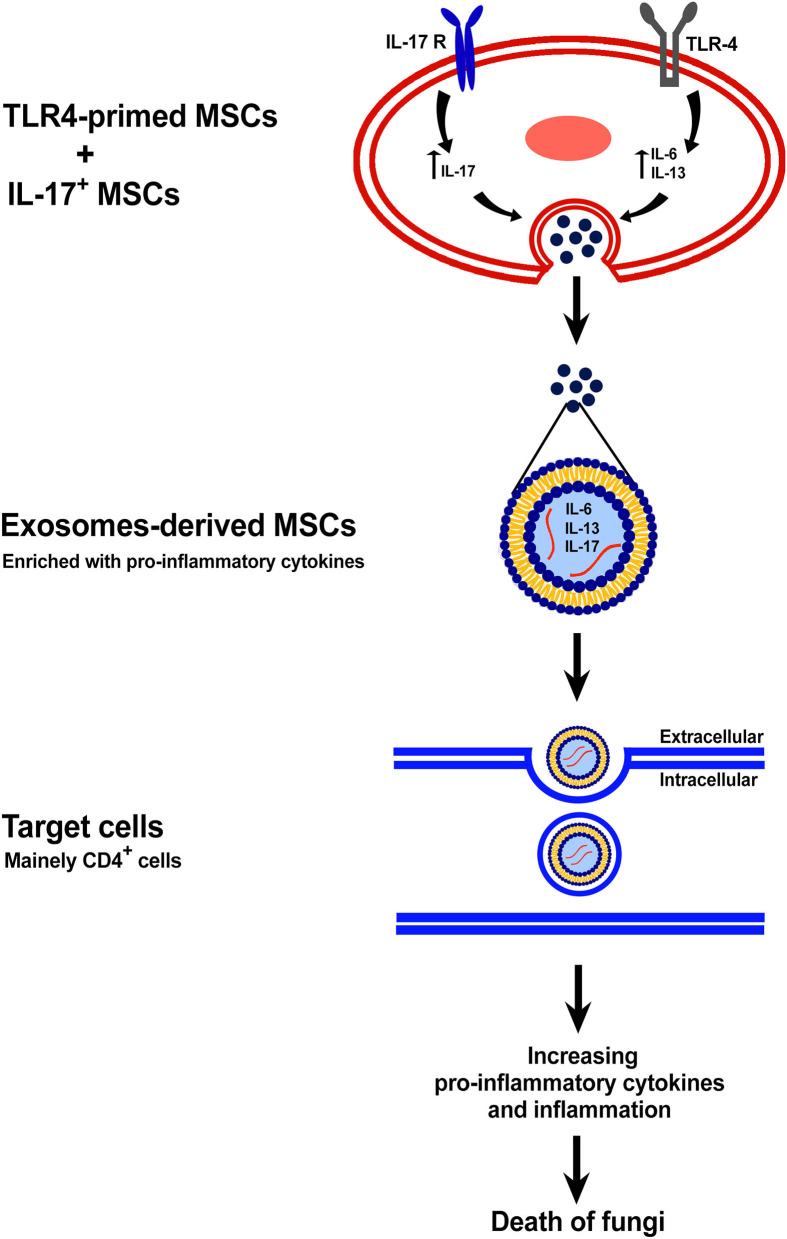
The exosome-derived TLR4-primed and IL-17^+^ MSCs. This figure shows the mechanism of anti-fungal effects of exosomes-derived new subtypes of MSCs.

However, some previous studies have shown that another type of MSCs has an immune-stimulatory effect, and this variety of the biological functions of MSCs depends on Toll-like receptors (TLRs) (Figure 1) (Waterman et al., 2010; Yang et al., 2013). It was shown that engagement of TLR-4 could enhance the production of pro-inflammatory mediators such as IL-17 and these MSCs are called TLR4-primed MSCs (Figure 1) (Waterman et al., 2010; Yang et al., 2013). In contrast, it was shown that TLR3-primed MSCs act as an immunomodulatory subset of MSCs (Waterman et al., 2010; Yang et al., 2013). The TLR4-primed MSCs, in contrast with TLR3-primed MSCs, was shown to increase expression of IL-6 and IL-13 as a pro-inflammatory cytokine and decrease IL-4, IDO, and PGE_2_ as an immunomodulatory cytokine and chemokine (Figure 1) (Waterman et al., 2010; Yang et al., 2013). IL-17 is a pro-inflammatory cytokine that plays a crucial role in intracellular and extracellular pathogenic defense (Yang et al., 2013; Schinocca et al., 2021). It was shown that a subpopulation of IL-17^+^ MSCs could inhibit *C. albicans* (Yang et al., 2013). Taken together, it might result that TLR4-primed and IL-17^+^ subsets of MSCs could be good candidates for fighting against fungal diseases (Figures 1, 2) (Waterman et al., 2010; Yang et al., 2013).

### The Extracellular Vesicles (EVs) and Its Classification

EVs have the main role in cell-to-cell communications (Andaloussi et al., 2013),and have been observed in both eukaryotes and prokaryotes (Ellis and Kuehn, 2010; Andaloussi et al., 2013). Studies have shown that the EVs could transfer the proteins and nucleic acids by its bilayer membrane (Lee et al., 2012; Ratajczak et al., 2012). Due to their potential for transferring proteins and nucleic acids, EVs are used widely as drug delivery agents (Elsharkasy et al., 2020). In order to best discuss the biological roles of EVs, here we describe the classification of EVs. The EVs based on their cellular origin, biological function, biogenesis, and size classified into three main groups: exosomes, microvesicles, and apoptotic bodies (Andaloussi et al., 2013; Yáñez et al., 2015). The two first particles, the exosomes and microvesicles, have been shown to have therapeutic effects (Wang et al., 2015; Phinney and Pittenger, 2017). The exosomes, with 40–120 nm in size, are generated by the endolysosomal pathway. In contrast with exosomes, the microvesicles are generated by budding from the cell surface (Andaloussi et al., 2013; Raposo and Stoorvogel, 2013). The exosomes with their non-sized particles, composed of a bilayer membrane and cytoplasm, contained mRNA, miRNA, and other RNAs' generated from the parent cell (Andaloussi et al., 2013; Raposo and Stoorvogel, 2013).

### The Exosomes-Derived MSCs and Their Biological Activity

Stem cells, especially mesenchymal stem cells, were used widely in past decades as a candidate for therapies of various diseases. In recent years, exosome-derived stem cells were substitutionally used for regenerative and immune-therapy as a cell-free therapy (Ji et al., 2019; Qiu et al., 2020). Previous studies have shown that the exosome-derived stem cells contained various bioactive molecules, especially proteins and microRNAs which originated from maternal cells (Baharlooi et al., 2020; Ma et al., 2020). These exosomes were shown to have some biological effects inherited from their maternal cells (Baharlooi et al., 2020). For instance, the exosome-derived MSCs displayed angiogenesis, regeneration, and especially anti-inflammatory effects (Baharlooi et al., 2020). Moreover, it was shown that these exosomes could carry various cytokines and chemokines originated and produced by the maternal cell (Di Trapani et al., 2016; Baharlooi et al., 2020). So, here we can hypothesize that the TLR4-primed MSCs could pass their pro-inflammatory cytokines and chemokines into exosomes derived from them. Exosomes-derived TLR4-primed MSCs could trigger the host immune system to start inflammation against fungal pathogens and fight against the immunosuppressive path of fungi.

## Discussion

The MSCs have been used in the treatment of microbial diseases for the past decades (Zhou and Xu, 2020). In most microbial diseases, the host-microbe interactions cause inflammation, which damaged host tissues (Qiu et al., 2020). Some of the subsets of MSCs, using the production of anti-inflammatory and immunomodulatory cytokines and chemokines, serve to downregulate the host immune system and reduce host tissue damages (Waterman et al., 2010; Baharlooi et al., 2020). That is why the MSCs were widely used in past decades for inflammatory and autoimmune diseases treatment. Among all microbial diseases, the pathogenesis of fungal diseases is more complicated. The fungi pathogen at the first stages of pathogenesis downregulates the immune system of the host body using TGF-β-transporting vesicles produced by induced monocytes (Netea et al., 2002; Halder et al., 2020). Using immunosuppression, the pathogen could survive better.

In recent years, it was noticed that the different subtypes of MSCs could show different biological activities (Waterman et al., 2010; Yang et al., 2013; Baharlooi et al., 2020). It was shown that induction of TLR-4 of MSCs could enhance its immune-stimulatory activity using the production of pro-inflammatory cytokines and chemokines (Waterman et al., 2010; Yang et al., 2013). As is obvious, in contrast with other microbial pathogenesis (Nauta and Fibbe, 2007) the fungal pathogen stops inflammation and downregulates the host immune system; so to fight that, the immune system needs to be upregulated and made able to inflame (Waterman et al., 2010; Yang et al., 2013). It was shown that the TLR4-primed and IL-17^+^ subsets of MSCs could express pro-inflammatory cytokines and chemokines, which could lead to inflammation (Waterman et al., 2010; Yang et al., 2013). These subtypes of MSCs could be an agent for fungal diseases treatment.

As is known, cell therapy has some challenges for human diseases therapy (Choi and Lee, 2016). The exosomes, as a cell-free therapy, solve most of the problems of cell therapy (Choi and Lee, 2016). Unlike a cell therapy, the exosomes are capable of crossing the blood-brain barrier and traveling through capillaries, and owing to their small sizes they are safe from reticuloendothelial system clearing (Li and Huang, 2009; Choi and Lee, 2016; Baharlooi et al., 2020). Moreover, as the exosomes inherited some of the molecules and biological activity of their maternal cells, they could be a good substitute for cell therapy (Di Trapani et al., 2016; Baharlooi et al., 2020; Ma et al., 2020). The exosome-derived MSCs showed to have anti-inflammatory and regenerative effects, the same as their maternal cells (Baharlooi et al., 2020). Several companies are developing exosome-derived products to take advantage of these applications, which suggests that in the future exosomes and their derived applications will be a viable choice for various disease therapies (Table 1).

**Table 1:** A list of companies producing various kinds of exosome-related products for therapeutic approaches.

**Product application(s)**	**Company**	**Web site**
Cancer detection	Exosomics	exosomics.eu
Cancer detection	Lonza	lonza.com
Carriers	Anjarium Biosciences	anjarium.com
Carriers	Codiak Biosciences	codiakbio.com
Carriers	Ilias Biologics Inc.	iliasbio.com
Carriers	MDimune	mdimune.com
Carriers	Tavec	tavecpharma.com
Exosome detection	NanoView Biosciences	nanoviewbio.com
Exosome isolation	Clara Biotech	clarabio.tech
Exosome isolation	EverZom	
Immunotherapy enhancer	EV Therapeutics	evtherapeutics.com
Inflammation therapy	The Cell Factory	esperite.com
Regenerative medicine	Aegle Therapeutics	aegletherapeutics.com
Regenerative medicine	Aruna Bio	arunabio.com
Regenerative medicine	Capricor Therapeutics	capricor.com
Regenerative medicine Vaccine	Ciloa	ciloa.fr
Regenerative medicine	Creative Medical Technologies Holdings	creativemedicaltechnology.com
Regenerative medicine	Direct Biologics	
Regenerative medicine	Evox Therapeutics	evoxtherapeutics.com
Regenerative medicine	Exocel Bio	exocelbio.com
Regenerative medicine	ExoCoBio	exocobio.com
Regenerative medicine	Exopharm	exopharm.com
Regenerative medicine	Exosome	exosomesciences.com
Regenerative medicine	Exogenus Therapeutics	exogenus-t.com
Regenerative medicine	Invitrx's	www.invitrx.com
Regenerative medicine	Kimera Labs	kimeralabs.com
Regenerative medicine	Oasis Diagnostics	4saliva.com
Regenerative medicine	OmniSpirant	omnispirant.com
Regenerative medicine	Organicell	organicell.com
Regenerative medicine	Percia Vista	perciavista.co
Regenerative medicine	Regen Suppliers	regensuppliers.com
Regenerative medicine	ReNeuron	reneuron.com
Regenerative medicine	RoosterBio	roosterbio.com
Regenerative medicine	Stem Cell Medicine Ltd.	stemcell-medicine.com
Regenerative medicine	Unicyte	unicyte.ch
Regenerative medicine	VivaZome Therapeutics	vivazome.com
Regenerative medicine	XOStem	xostem.com
Tumor exosome capture	Aethlon Medical	aethlonmedical.com

As the maternal cell produces anti-inflammatory cytokines and chemokines, these molecules could pass into the exosomes (Wang et al., 2015; Baharlooi et al., 2020). Based on previous results, it could be hypothesized that the TLR4-primed and IL-17^+^ subsets of MSCs could pass its produced pro-inflammatory cytokines and its immune-stimulatory activity into its exosomes. These exosomes could be a treatment for fungal pathogenesis.

During the past decade, many preclinical studies of exosomes have been conducted. Some of these studies have been shown in Table 2. These studies demonstrated that exosomes-derived MSCs could have anti-inflammatory, anti-atopic dermatitis, anti-neurodegenerative, anti-liver fibrosis biological activities, and so on (Li et al., 2013; Cho et al., 2018; Lee et al., 2018; Gowen et al., 2020). Despite many preclinical studies of exosomes, clinical studies of the MSCs-derived exosomes are few (Gowen et al., 2020). The MSCs-derived exosomes were used in previous clinical studies to treat diseases such as graft-versus-host disease (Kordelas et al., 2014), chronic kidney disease with grade III and IV (Nassar et al., 2016), type II diabetes (Sun et al., 2018), and prevention of the onset of type-1 diabetes *via* suppression of immune system and induction of beta cells regeneration (Ezquer et al., 2012). There are also several studies which have not yet been published.

**Table 2:** Animal studies of exosomes-derived MSCs.

**Cell source**	**Therapeutics**	**Transplantation**	**Donor species**	**Recipient species**	**Biological effects**	**References**
Embryonic MSCs	Exosome	Xenotransplant	Human	Rat	Osteochondral regeneration promotion	Zhang et al., 2016
Adipose tissue-derived MSCs	Exosome	Xenotransplant	Human	Mouse	Atopic dermatitis alleviation	Cho et al., 2018
Adipose tissue-derived MSCs	Exosome	Xenotransplant	Human	Rat	Evaluation of exosomes cell toxicity	Ha et al., 2020
Bone marrow- derived MSCs	Exosome	Xenotransplant	Rat	Mouse	Neuroprotective effect *via* inhibiting early neuroinflammation	Ni et al., 2019
Wharton's jelly-derived MSCs	Exosome	Xenotransplant	Human	Rat	Anti-inflammatory effects on microglia in perinatal brain injury	Thomi et al., 2019
Umbilical cord-derived MSCs	Exosome	Xenotransplant	Human	Mouse	Acute liver failure alleviation	Jiang et al., 2019
Bone marrow- derived MSCs	Exosome	Xenotransplant	Rat	Mouse	Inadequate promotion of bone regeneration in type 1 diabetes	Zhu et al., 2019
Bone marrow- derived MSCs	Exosome	Allotransplant	Rabbit	Rabbit	Regulation of injured endometrium repair	Yao et al., 2019
Umbilical cord-derived MSCs	Exosome	Xenotransplant	Human	Mouse	Inflammatory bowel disease treatment	Mao et al., 2017
Adipose tissue-derived MSCs	Exosome	Allotransplant	Rat	Rat	Promotion of endometrium regeneration in rats with intrauterine adhesion	Zhao et al., 2020
Placental- derived MSCs	Exosome	Xenotransplant	Human	Mouse	Enhancement of angiogenesis and improvement of neurologic function	Zhang et al., 2020b
Umbilical cord-derived MSCs	Exosome	Xenotransplant	Human	Mouse	Inhibition of silica-induced PF and improve lung function	Xu et al., 2020a
Bone marrow- derived MSCs Adipose tissue-derived MSCs	Exosome	–	–	Rat	Improvement of erectile dysfunction in bilateral cavernous nerve injury	Li et al., 2018
Bone marrow- derived MSCs	Exosome	Allotransplant	Rat	Rat	Rescuing myocardial ischaemia/reperfusion injury	Liu et al., 2017
Umbilical cord-derived MSCs	Exosome	Xenotransplant	Human	Rat	Inhibition of vein graft neointimal hyperplasia and acceleration of reendothelialization	Qu et al., 2020
Adipose tissue-derived MSCs	Exosome	Allotransplant	Mouse	Mouse	Exo-circAkap7, a potential treatment for cerebral ischemic injury.	Xu et al., 2020b
Bone marrow- derived MSCs	Exosome	Xenotransplant	Rat	Guinea pig	Reduction of demyelination and neuroinflammation in an immune-induced demyelination model	Li et al., 2019
Bone marrow- derived MSCs	Exosome	Allotransplant	Rat	Rat	Promotion of immunotolerance and prolong the survival of cardiac allografts	He et al., 2018

*MSCs, mesenchymal stem cells*.

However, stem cell-derived exosomes have some limitations for clinical studies. For instance, large-scale exosome production is lacking; large-scale exosome quantifications methods with rapid and accurate results, and determination of exosomes' contents with high accuracy also present dificulties (Gowen et al., 2020). Moreover, the pharmacokinetics, pathways, targets and mechanisms of action of the exosomes in the human body still remain unknown. Additionally, more studies are needed to evaluate the correct dosage of the exosomes for clinical studies in order to prevent possible toxicities (Gowen et al., 2020).

## Author Contributions

SOG: data collection, manuscript writing, idea conception, study design, and approved the final version.

## Conflict of Interest

The author declares that the research was conducted in the absence of any commercial or financial relationships that could be construed as a potential conflict of interest.

## Publisher's Note

All claims expressed in this article are solely those of the authors and do not necessarily represent those of their affiliated organizations, or those of the publisher, the editors and the reviewers. Any product that may be evaluated in this article, or claim that may be made by its manufacturer, is not guaranteed or endorsed by the publisher.
